# Limited Ability of Posaconazole To Cure both Acute and Chronic Trypanosoma cruzi Infections Revealed by Highly Sensitive *In Vivo* Imaging

**DOI:** 10.1128/AAC.00520-15

**Published:** 2015-07-16

**Authors:** Amanda Fortes Francisco, Michael D. Lewis, Shiromani Jayawardhana, Martin C. Taylor, Eric Chatelain, John M. Kelly

**Affiliations:** a Department of Pathogen Molecular Biology, London School of Hygiene and Tropical Medicine, London, United Kingdom; b Drugs for Neglected Diseases Initiative, Geneva, Switzerland

## Abstract

The antifungal drug posaconazole has shown significant activity against Trypanosoma cruzi *in vitro* and in experimental murine models. Despite this, in a recent clinical trial it displayed limited curative potential. Drug testing is problematic in experimental Chagas disease because of difficulties in demonstrating sterile cure, particularly during the chronic stage of infection when parasite burden is extremely low and tissue distribution is ill defined. To better assess posaconazole efficacy against acute and chronic Chagas disease, we have exploited a highly sensitive bioluminescence imaging system which generates data with greater accuracy than other methods, including PCR-based approaches. Mice inoculated with bioluminescent T. cruzi were assessed by *in vivo* and *ex vivo* imaging, with cyclophosphamide-induced immunosuppression used to enhance the detection of relapse. Posaconazole was found to be significantly inferior to benznidazole as a treatment for both acute and chronic T. cruzi infections. Whereas 20 days treatment with benznidazole was 100% successful in achieving sterile cure, posaconazole failed in almost all cases. Treatment of chronic infections with posaconazole did however significantly reduce infection-induced splenomegaly, even in the absence of parasitological cure. The imaging-based screening system also revealed that adipose tissue is a major site of recrudescence in mice treated with posaconazole in the acute, but not the chronic stage of infection. This *in vivo* screening model for Chagas disease is predictive, reproducible and adaptable to diverse treatment schedules. It should provide greater assurance that drugs are not advanced prematurely into clinical trial.

## INTRODUCTION

Chagas disease is a major public health problem in Latin America and is increasingly prevalent in other regions as a result of migration patterns (1, 2). The causative agent, Trypanosoma cruzi, is transmitted to humans predominantly by hematophagous triatomine bugs, although other routes include contaminated food and drink, blood transfusion and congenital transmission. After infection, patients progress to the acute stage of the disease, where parasites are readily detectable in the bloodstream by microscopic examination. In most individuals, immune-mediated responses suppress parasitemia within 4 to 6 weeks and the majority of patients then remain asymptomatic, despite a lifelong low-level infection. However, years or often decades later, ca. 30% of those infected develop chronic Chagas disease pathology, typically cardiomyopathy and/or digestive megasyndromes (3). Because of the complex and long-term course of the infection, vaccine development is considered to be extremely challenging, and most biomedical research has focused on improving chemotherapy.

For the last 40 years, the nitroheterocyclic compounds benznidazole and nifurtimox have been the only drugs available to treat Chagas disease (4, 5). This is despite the fact that therapeutic schedules are long, treatment failures have been frequently reported, and both drugs exhibit toxicity. In addition, their efficacy in preventing or alleviating chronic disease pathology remains to be conclusively demonstrated (6, 7). Benznidazole and nifurtimox are prodrugs and both are activated within T. cruzi by the same mitochondrial nitroreductase (TcNTR) (8), leading to the generation of reactive metabolites which mediate parasite killing (9–11). This shared activation mechanism provides potential for cross-resistance (8, 12, 13) and highlights the need to identify additional therapeutic agents which target distinct biochemical pathways. In this context, sterol metabolism in T. cruzi has generated considerable interest, particularly the enzymes involved in ergosterol biosynthesis (14, 15). The antifungal drug posaconazole for example, is a potent inhibitor of the T. cruzi sterol 14α-demethylase (CYP51) and has shown significant antiparasitic activity *in vitro* and *in vivo* (16–18). Furthermore, combination therapy with benznidazole has demonstrated that murine infections can be cured with a reduced dosing regime (19, 20). However, in a recent randomized clinical trial against chronic T. cruzi infection, posaconazole was shown to have limited curative potential (21), and *in vitro* studies have found it to be less active than benznidazole (22).

The vast majority of Chagas disease patients are only diagnosed once they begin to display chronic disease pathology or after testing prior to blood donation or surgical procedures. Drug trials against chronic stage infections are particularly challenging because it is difficult to unequivocally demonstrate sterile cure. In addition, lack of knowledge of the sites of parasite persistence can be a confounding factor that impacts on the reproducibility of PCR-based methodologies, making it difficult to accurately assess parasite burden in real time. To streamline the drug discovery process, we sought to improve the utility of current predictive models of experimental Chagas disease by developing an enhanced *in vivo* imaging system. This was achieved by engineering trypanosomes to express a red-shifted luciferase reporter that emits tissue-penetrating orange-red light (λ_em_ 617 nm) (23, 24). In T. cruzi, the bioluminescent reporter is expressed at similar levels in different parasite life cycle stages, has no effect on growth properties or virulence, and is maintained at constant levels for more than 12 months in the absence of selective drug pressure. Importantly, this *in vivo* imaging system has a limit of detection of between 100 and 1,000 parasites and has allowed parasite burden to be assessed in real time during experimental chronic infections in individual mice (24). Throughout chronic infections, dynamic bioluminescence foci can appear and disappear over a period of less than 24 h (24), consistent with a scenario where infected cells are being trafficked to and from peripheral sites. In BALB/c mice infected with the CL Brener strain, the gastrointestinal tract was found to be the major site of parasite persistence. Unexpectedly, in this model, infection of the heart was rarely observed in the chronic stage, even though these mice continued to exhibit cardiac inflammation and diffuse fibrosis, signatures of chronic Chagas disease pathology.

The enhanced sensitivity of this red-shifted luciferase based reporter system has the potential to provide new approaches for monitoring the effectiveness of drugs against experimental Chagas disease and should be a valuable addition to the drug discovery pipeline. Here, we describe its use to assess the efficacy of posaconazole to treat acute and chronic experimental infections. In line with a recent clinical trial, our predictive model suggests major limitations in the utility of this drug.

## MATERIALS AND METHODS

### Mice and infections.

Female BALB/c mice were purchased from Charles River (United Kingdom) and CB17 SCID mice were bred in-house. Animals were maintained under specific-pathogen-free conditions in individually ventilated cages, where they experienced a 12-h light/dark cycle and had access to food and water *ad libitum*. All experiments were carried out under UK Home Office license PPL 70/6997 and approved by the LSHTM Animal Welfare and Ethical Review Board. Mice were aged 8 to 12 weeks when infected with a bioluminescent reporter clone derived from the genome reference strain CL Brener (24). In standard experiments, 10^4^ *in vitro*-derived tissue-culture trypomastigotes or thawed cryopreserved bloodstream trypomastigotes (BTs) in 0.2 ml of phosphate-buffered saline (PBS) were first used to infect SCID mice via intraperitoneal (i.p.) inoculation. Parasitemic blood from these SCID mice was obtained 2 to 3 weeks later and adjusted to 5 × 10^3^ BTs/ml with PBS. BALB/c mice were then infected with 10^3^ BTs via i.p. injection (24).

### Treatment.

For drug treatment, benznidazole (Hoffmann-La Roche AG) was prepared from powder at 10 mg/ml in 7% Tween 80, 3% ethanol (vol/vol), and 90% (vol/vol) water. Posaconazole (Sequoia Research Products, Ltd.) was prepared at 2 mg/ml in 5% (vol/vol) dimethyl sulfoxide and 95% (vol/vol) HPMC-SV (0.5% [wt/vol] hydroxypropyl methylcellulose, 0.5% [vol/vol] benzyl alcohol and 0.4% [vol/vol] Tween 80). Noxafil (MSD, Ltd.), a liquid formulation of posaconazole (40 mg/ml), was diluted to 2 mg/ml in water. Mice were treated with standard doses of benznidazole (100 mg/kg/day) or posaconazole (20 mg/kg/day) by oral gavage for consecutive days, as required. To facilitate the detection of residual infection after treatment, BALB/c mice were immunosuppressed in some experiments with cyclophosphamide (200 mg/kg) by i.p. injection at 3- to 4-day intervals, for a maximum of three doses.

### *In vivo* bioluminescence imaging.

Mice were injected i.p. with 150 mg of d-luciferin (Perkin-Elmer)/kg in Dulbecco Ca^2+^/Mg^2+^-free PBS and then anesthetized using 2.5% (vol/vol) gaseous isoflurane in oxygen. To measure bioluminescence, mice were placed in an IVIS Lumina II system (Caliper Life Science), and both dorsal and ventral images were acquired 10 to 20 min after d-luciferin administration using LivingImage 4.3. Exposure times varied between 30 s and 5 min, depending on signal intensity. Anesthesia was maintained throughout via individual nose cones. After imaging was complete, the mice were revived and returned to cages. To estimate the parasite burden, whole-body regions of interest were drawn using LivingImage v4.3 to quantify bioluminescence expressed as total flux (photons/second [p/s]). The detection threshold was established previously using data from control uninfected mice (24). Animals where bioluminescence intensity was consistently below 5 × 10^3^ p/s/sr/cm^2^ in both dorsal and ventral images following immunosuppression, were regarded as cured, subject to confirmation by *ex vivo* assessment (below).

### Assessment of treatment efficacy by *ex vivo* imaging.

**FIG 1:**
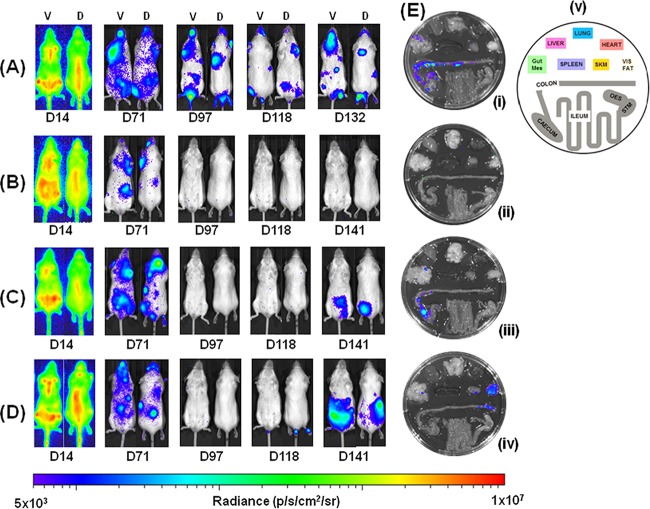
Benznidazole, but not posaconazole, cures mice chronically infected with T. cruzi. Mice infected with bioluminescent T. cruzi were injected with 150 mg of d-luciferin/kg, anesthetized, and imaged using an IVIS Lumina II system (Materials and Methods). (A to D) Ventral (V) and dorsal (D) images of individual representative infected mice. (A) Untreated mouse; (B) mouse treated with benznidazole at 100 mg/kg on days 74 to 93 postinfection then immunosuppressed by cyclophosphamide treatment (200 mg/kg) on days 113, 118, and 128; (C) mouse treated with posaconazole (Noxafil formulation) at 20 mg/kg on days 74 to 93 and then immunosuppressed as described above; (D) mouse treated with posaconazole (HPMC-SV formulation) on days 74 to 93 and immunosuppressed as described above. (E) Tissue-specific *ex vivo* imaging. (i) Untreated mouse at 132 dpi; (ii) mouse at 147 dpi, which had been treated with benznidazole, and then immunosuppressed, as described above; (iii and iv) mice at 147 and 148 dpi, which had been treated with posaconazole (Noxafil and HPMC-SV formulations, respectively) and then immunosuppressed as described above. (v) Schematic that identifies the positions of organs displayed in panels i to iv. Gut Mes, gut mesentery tissue; OES, esophagus; SKM, skeletal muscle; STM, stomach; VIS FAT, visceral fat/adipose tissue. The heat map is on a log_10_ scale and indicates the intensity of bioluminescence from low (blue) to high (red); the minimum and maximum radiances for the pseudocolor scale are shown.

Selected organs and tissue samples from all mice were assessed for infection by *ex vivo* imaging (Fig. 1E), as described previously (24). Briefly, mice were injected with 150 mg of d-luciferin/kg i.p. and then sacrificed by exsanguination under terminal anesthesia 7 min later. Mice were perfused with 10 ml of d-luciferin at 0.3 mg/ml in PBS via the heart. Organs and tissues were excised, transferred to a petri dish or culture dish, soaked in 0.3 mg of d-luciferin/ml in PBS, and then imaged as for the live mice. Routinely, the rest of the carcass was also assessed for bioluminescence associated with skin, skeletal muscle, or remaining adipose tissue. As with *in vivo* imaging, a bioluminescence intensity of 5 × 10^3^ p/s/sr/cm^2^ was used as the threshold to designate cure.

### PCR-based detection.

Heart, large intestine, and blood tissues were snap-frozen on dry ice and stored at −80°C until required for DNA extraction. In the case of the gut, three 1-cm sections were pooled from the proximal colon, the midcolon region, and the rectum of each mouse. Samples were then thawed and immediately homogenized in at least 200 μl of lysis buffer (4 M urea, 200 mM Tris, 20 mM NaCl, 200 mM EDTA [pH 7.4]) per 50 mg of tissue, using a BulletBlender Storm instrument (Next Advance). Proteinase K (Sigma) was added to the tissue suspension at 0.6 mg per 200 μl and incubated at 56°C for 1 h and then at 37°C overnight. DNA was extracted from lysates using a HighPure PCR template preparation kit (Roche) according to the manufacturer's instructions. Real-time PCRs were prepared using a QuantiTect SYBR green PCR kit (Qiagen) and analyzed using a RotorGene 3000 instrument. Each reaction mixture contained 50 ng of DNA and 0.5 μM concentrations of each primer. The T. cruzi-specific primers TCZ-F and TCZ-R (25) targeting the 195-bp satellite repeat (10^4^ copies in the CL Brener genome) or the mouse-specific primers GAPDHf and GAPDHr (26) targeting the murine *gapdh* gene were used.

T. cruzi-specific quantitative PCR (qPCR) threshold cycle (*C_T_*) values were converted to inferred numbers of parasite equivalents (p.e.) by reference to a standard curve with a range of 2.5 × 10^6^ to 2.5 × 10^−1^ p.e./ml of tissue lysate. The T. cruzi standard curve was established from serial dilution of a DNA sample derived from 75 mg of homogenized muscle tissue, spiked with 2 × 10^7^ epimastigotes, using DNA from unspiked equivalent samples as the diluent. The murine DNA content was determined by normalizing mouse-specific qPCR *C_T_* values by reference to a standard curve with a range of 2.5 × 10^1^ to 2.5 × 10^−4^ μg/ml. The murine standard curve was established from serial dilution of a mouse DNA sample using herring sperm DNA as the diluent. Due to the nonspecific fluorescence inherent to this SYBR green qPCR method, we defined parasite detection limits as means plus three standard deviations (SD) for samples from uninfected control mice.

### Statistics.

Results are shown as means ± the SD (or standard errors of the mean) where sample sizes are equal or unequal, respectively. Individual animals were used as the unit of analysis for *in vivo* and *ex vivo* experiments. For spleen mass, means were compared using a Student *t* test.

## RESULTS

### Benznidazole and posaconazole efficacy against chronic-stage T. cruzi infections.

**FIG 2:**
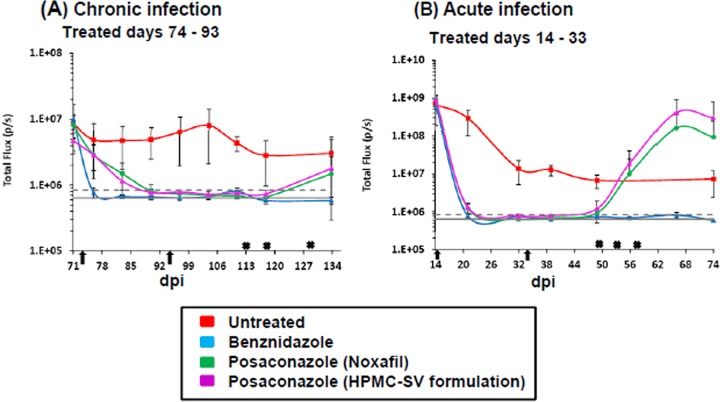
Quantification of whole animal bioluminescence (ventral and dorsal) after treatment with benznidazole and posaconazole. (A) Infected mice treated starting 74 dpi for 20 days by the oral route. (B) Mice treated starting 14 dpi for 20 days by the oral route. Red squares, untreated mice (*n* = 5, acute; *n* = 6, chronic); blue squares, treated with benznidazole at 100 mg/kg (*n* = 10); green squares, treated with posaconazole (Noxafil) at 20 mg/kg (*n* = 9); purple squares, treated with posaconazole (HPMC-SV formulation) at 20 mg/kg (*n* = 10). Arrows indicate the start and endpoints of treatment. Gray lines indicate detection threshold determined as the mean (solid line) and mean plus 2 SD (dashed line) of background bioluminescence of control uninfected mice. Crosses signify the dates of cyclophosphamide treatment (200 mg/kg). Inoculation failed to result in an infection in one mouse in each of the acute and chronic infection studies. These mice were not treated (Noxafil cohort) and excluded from the analysis.

BALB/c mice, infected i.p. with 10^3^ bioluminescent bloodstream-form T. cruzi trypomastigotes (CL Brener strain), were monitored by *in vivo* imaging (Fig. 1A; see also Materials and Methods). In this experimental model, peak parasitemia occurs after 14 days and is followed by an immune-mediated reduction in parasite load during progress to the chronic stage at 40 to 50 days postinfection (dpi) (Fig. 2B) (24). After 74 days, cohorts of mice were treated daily for 20 days by the oral route with benznidazole (100 mg/kg) or with one of two posaconazole formulations (20 mg/kg). These dosing regimes have been widely used for experimental purposes (19, 20, 27, 28). Benznidazole acted rapidly and the whole-body bioluminescence of each mouse fell to undetectable levels within 5 days (Fig. 1B and 2A). Posaconazole was slower acting, but by the conclusion of the treatment period the inferred parasite load had also dropped to background levels. The bioluminescence profile during treatment was very similar with both posaconazole formulations (Fig. 1C and D and Fig. 2A). At 20 days after the cessation of treatment (113 dpi), half the mice in each cohort were immunosuppressed (Materials and Methods). No signs of infection were observed in any of the benznidazole-treated mice in either the immunosuppressed or the immunocompetent groups (Fig. 1B). However, in the posaconazole-treated group, the infection relapsed in all of the cyclophosphamide-treated mice (Fig. 1C and D and Fig. 2A; Table 1).

**TABLE 1:** Summary of drug efficacy against acute and chronic T. cruzi infections inferred from bioluminescence^*a*^

Drug	Disease state	Treatment time (days)	Daily dose (mg/kg)	No. cured/no. tested
Benznidazole	Chronic	20	100	5/5
Benznidazole	Acute	20	100	5/5
Benznidazole	Chronic	10	100	6/6
Benznidazole	Chronic	5	100	6/6
Posaconazole	Chronic	20	20	0/9
Posaconazole	Acute	20	20	3/19

a Data were collated from the experiments illustrated in Fig. 1, 4, and 6. Mice were designated as cured only when bioluminescence negative by both *in vivo* and *ex vivo* imaging following immunosuppression (see Materials and Methods). In the posaconazole treatment, data from both formulations were pooled.

Organs from all of the mice were assessed for infection by *ex vivo* imaging at the experimental endpoint (Fig. 1E; see also Materials and Methods). In accordance with experiments using this and other mouse-parasite combinations (24; M. D. Lewis, unpublished observations), persistent bioluminescent foci at this point of the infection (∼148 dpi) were associated predominantly with the gastrointestinal tract (mainly the colon and stomach) in untreated mice and only sporadically with other major organs/tissues. Mice were considered cured if they were bioluminescence negative by both *in vivo* and *ex vivo* imaging, following cyclophosphamide treatment (see Materials and Methods). Based on these criteria, none of the nine chronically infected mice treated with posaconazole and subsequently immunosuppressed were cured. In contrast, all five mice that were treated with benznidazole and then immunosuppressed were inferred to be parasite-free.

**FIG 3:**
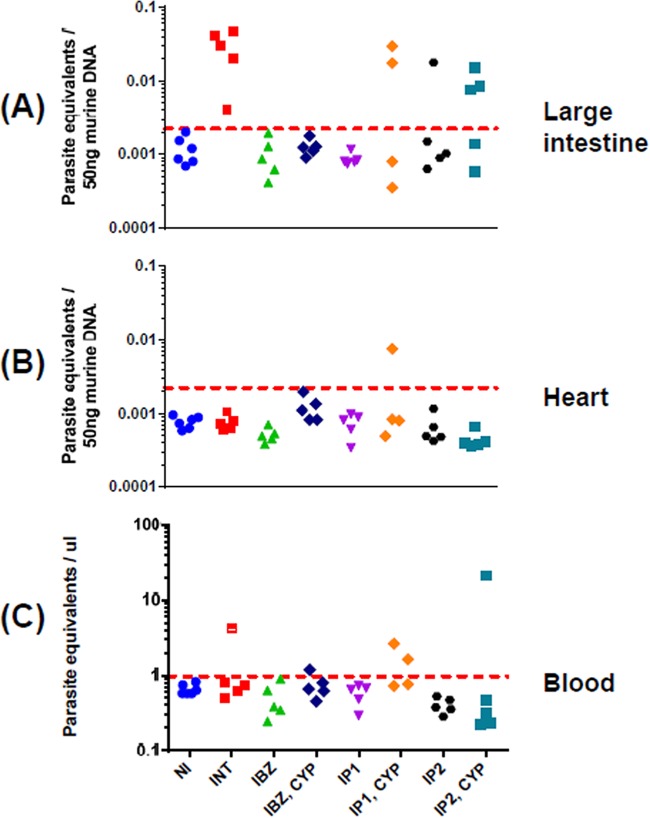
qPCR-inferred parasite loads in drug-treated chronically infected mice. DNA was extracted from large intestine (A), heart (B), and blood (C) samples, and the relative amounts of T. cruzi and murine DNA were quantified by real-time PCR amplification of the multicopy 195-bp satellite repeat and of the *gapdh* gene, respectively (Materials and Methods). The limit of detection is represented by the dotted line which corresponds to the mean plus 3 SD of large intestine, heart, or blood samples from uninfected mice. Above this line, there is a linear correlation with the parasite number, as established by a standard curve (see Materials and Methods). In panels A and B, the baseline is equivalent to <1 parasite per 5 million murine cells. In panel C, the baseline established with the multicopy 195-bp repeat corresponds to 1 parasite equivalent per μl of blood. NI, not infected; INT, infected nontreated; IBZ, infected, benznidazole treated (as in Fig. 1); “IBZ, CYP,” infected, benznidazole treated, and then immunosuppressed with cyclophosphamide; IP1, infected, posaconazole (Noxafil) treated; “IP1, CYP,” infected, posaconazole (Noxafil) treated, and then immunosupressed with cyclophosphamide; IP2, infected, posaconazole (HPMC-SV formulation) treated; “IP2, CYP,” infected, posaconazole (HPMC-SV formulation) treated, and then immunosuppressed.

qPCR after immunosuppression has until now been the most accurate technique for defining parasitological cure in T. cruzi infections (20, 28, 29). However, when we assessed the efficacy of this method in our chronic infection model, we found that PCR methodology had a tendency to overestimate the cure rate, particularly with posaconazole treatment. In chronically infected untreated mice, pooled gut tissues (see Materials and Methods) were PCR positive in each case and negative when mice were treated with benznidazole for 20 days, including the group that was subsequently immunosuppressed (Fig. 3A). With the posaconazole-treated nonimmunosuppressed mice, gut tissue was PCR negative in 9 of 10 cases, a finding also consistent with a high rate of cure. This inferred cure rate was reduced when tissues derived from mice that had also been cyclophosphamide treated were analyzed. The number of PCR-negative (cured) animals fell to 4 of 9, indicating that some low-level infections only become detectable by PCR after immunosuppression. However, even this reduced cure rate is at odds with data from bioluminescence imaging, which demonstrated unequivocally that posaconazole failed to eradicate parasites in any of the treated mice (Fig. 1, Table 1). In the case of cardiac tissue, with one exception, the results were PCR negative in all cases (Fig. 3B), in accordance with bioluminescence imaging of chronic-stage infections (Fig. 1) (25). When blood samples were analyzed, they were predominantly negative, even when the mice were untreated (Fig. 3C). Collectively, these results highlight the limitations of using PCR-based approaches to define parasitological cure in this dynamic chronic-stage model. The low level and sporadic nature of bloodstream parasitemia and the focal and highly dynamic nature of tissue infection, even within the gastrointestinal tract, appear to be the confounding factors which result in an overestimation of the cure rate when determined by qPCR alone.

**FIG 4:**
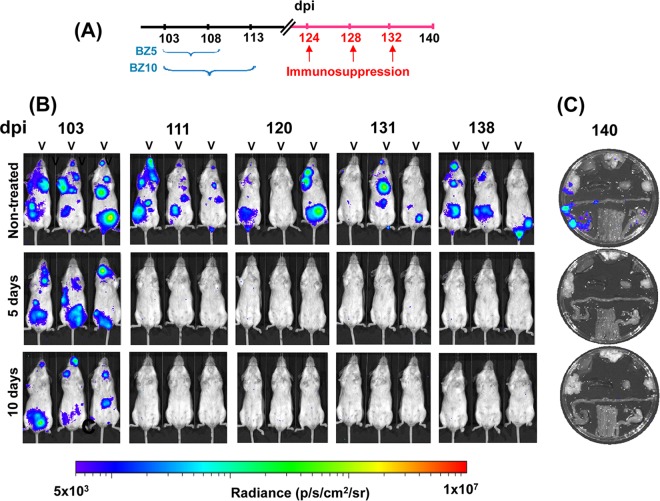
Assessing the ability of benznidazole to cure mice chronically infected with T. cruzi using 5- and 10-day treatment regimes. (A) Outline of experimental protocol. Cohorts of six mice were infected with bioluminescent T. cruzi (see Materials and Methods). At 103 dpi, the mice were treated with benznidazole (daily by the oral route, 100 mg/kg) for 5 or 10 days. As indicated, the mice were subsequently immunosuppressed with three i.p. doses of cyclophosphamide (200 mg/kg). (B) Ventral images of three representative mice from each cohort taken at the indicated day postinfection. (C) Representative *ex vivo* imaging of organs from infected mice at 140 dpi (see Materials and Methods). Organs are displayed in accordance with the schematic in Fig. 1Ev. Heat maps indicate the intensity of bioluminescence from low (blue) to high (red) (log_10_ scales); the minimum and maximum radiances for the pseudocolor scale are shown.

To further assess the ability of benznidazole to cure chronically infected mice, we reduced the treatment period to 10 and 5 oral doses (100 mg/kg/day) over consecutive days. In each case, bioluminescence fell below the level of detection by the completion of treatment, and subsequent immunosuppression of these mice did not lead to a relapse, as assessed by either *in vivo* or *ex vivo* imaging (Fig. 4). In this experimental model, therefore, there is scope to reduce the length of benznidazole treatment of chronic T. cruzi infections and still achieve a curative outcome.

**FIG 5:**
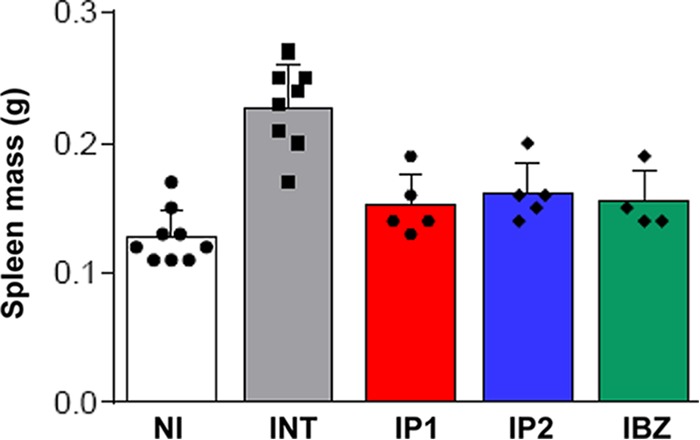
Effect of chronic T. cruzi infection and drug treatment on spleen mass. At 74 dpi, mice were treated with benznidazole (100 mg/kg) (IBZ) or posaconazole (20 mg/kg) (IP1 and IP2 represent the Noxafil and HPMC-SV formulations, respectively) by the oral route, daily for 20 days (see also Fig. 1). Spleens were harvested and weighed at the end of the experiment. Spleen weights in infected, nontreated mice (INT) were significantly greater than those in noninfected mice (NI) (*P* < 0.0001) or infected mice which had been treated (*P* < 0.005, in each case).

Splenomegaly is frequently observed in both acute and chronic experimental T. cruzi infections. Here, we observed that the spleens of chronically infected mice were approximately twice the mass of those from uninfected mice (Fig. 5). This spleen enlargement could be reversed by curative treatment with benznidazole (assessed by *in vivo* and *ex vivo* imaging, with an immunosuppressed group in parallel; Fig. 1). Interestingly, there was also a reversal of splenomegaly after posaconazole treatment. In these mice, there was a major reduction in parasite burden, but a curative outcome was not achieved (Fig. 1). Therefore, splenomegaly in this model is linked with parasite load and can be largely reversed, at least in the short term, without having to achieve a sterile cure.

### Benznidazole and posaconazole efficacy against acute-stage T. cruzi infections.

**FIG 6:**
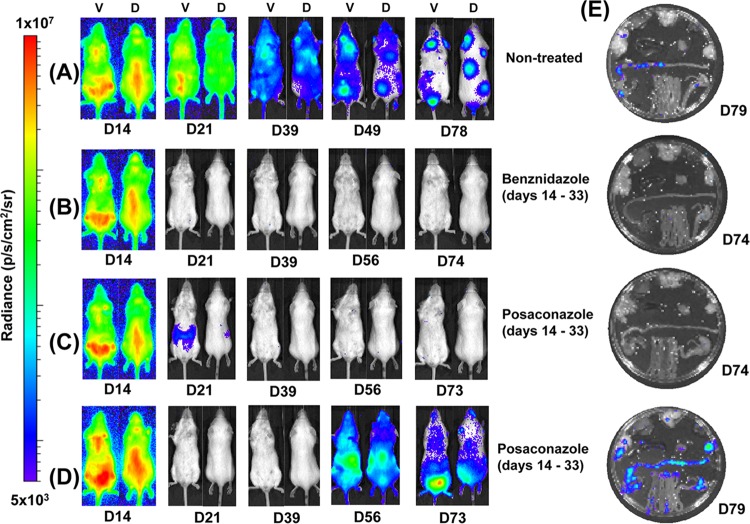
Posaconazole has limited efficacy as a treatment for acute T. cruzi infections. Mice (*n* = 10) were infected with bioluminescent T. cruzi (Fig. 1; see also Materials and Methods) and treatment initiated at the peak of the acute stage, day 14. (A to D) Ventral (V) and dorsal (D) images of representative individual mice. (A) Infected, nontreated. (B) Treated with benznidazole at 100 mg/kg on days 14 to 33 postinfection and then immunosuppressed by 200 mg/kg cyclophosphamide treatment on days 49, 53, and 57. (C) Treated and cured with posaconazole (20 mg/kg; Noxafil formulation) on days 14 to 33 and immunosuppressed as described above. (D) Treated with posaconazole (Noxafil formulation) on days 14 to 33 and immunosuppressed as described above. A total of 16 of 19 posaconazole-treated mice were assessed as noncured. One mouse did not become infected and was excluded from the study. (E) *Ex vivo* imaging of organs and tissues obtained from mice on days 74 to 79, as indicated, after drug treatment and immunosuppression. Organs and tissues were arranged as in Fig. 1Ev. Heat-maps are on log_10_ scales and indicate the intensity of bioluminescence from low (blue) to high (red).

Using the same experimental model as described above, we compared the ability of benznidazole and posaconazole to cure acute-stage T. cruzi infections. Treatment was started at the peak of parasite burden (14 dpi) with standard oral doses (benznidazole, 100 mg/kg; posaconazole, 20 mg/kg) administered daily for 20 days. Similar to the chronic-stage infections, benznidazole treatment resulted in a rapid fall in parasite load, with bioluminescence reduced to background levels within 5 days (Fig. 2B and 6B). There was no relapse of infection, when mice were assessed by *in vivo* or *ex vivo* imaging following immunosuppression (Fig. 6B and E), and all mice treated with benznidazole were therefore considered cured.

With posaconazole treatment, the reduction in bioluminescence was much more rapid than had been observed with chronic-stage infections (compare Fig. 2A and B), and only marginally slower than with benznidazole. Again, there were no significant differences in the efficacy of the two posaconazole formulations. Bioluminescence remained close to, or only slightly above background levels, until the mice were treated with cyclophosphamide (initiated 49 dpi) (Fig. 2B and 6). At this point, there was a rapid rebound in parasite load in most cases, with 16 of 19 mice displaying a clear bioluminescence signal (Fig. 6, Table 1). Of the three mice judged to be cured, one had been treated with the Noxafil and two had been treated with the HPMC-SV posaconazole formulation (see Materials and Methods). These results therefore suggest that although posaconazole is more effective at reducing the parasite load during the acute stage than it is during the chronic stage, it has only a limited ability to achieve a sterile cure in this experimental model, in either stage of the disease.

**FIG 7:**
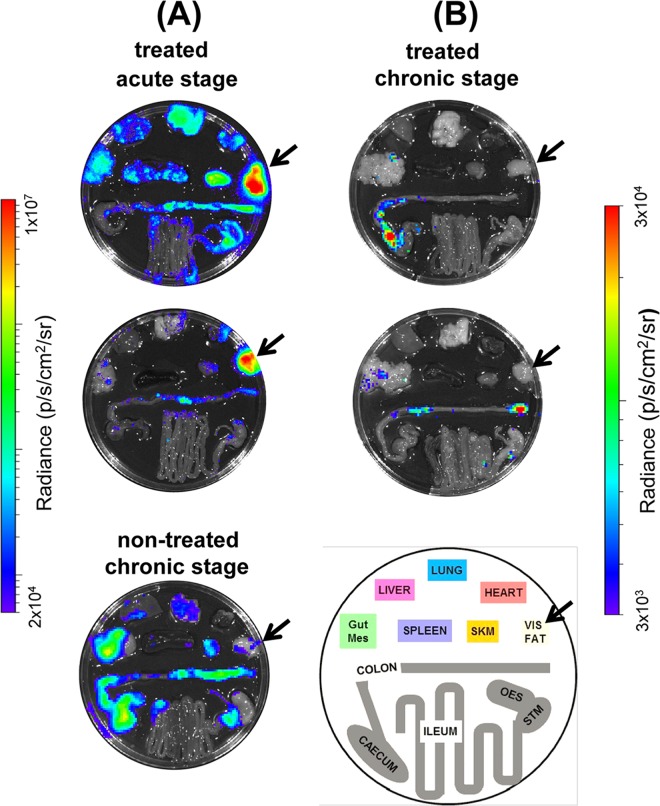
*Ex vivo* imaging of cyclophosphamide-induced parasite recrudescence after posaconazole treatment of mice in the acute and chronic stages of infection. (A) Mice in the acute stage of T. cruzi infection were treated with posaconazole for 20 days and then treated with cyclophosphamide (see legend to Fig. 6 for details). Images were taken 74 dpi. For comparison, the lower image shows parasite recrudescence after immunosuppression of a nontreated, chronically infected mouse (imaged at 173 dpi). (B) T. cruzi-infected mice, treated with posaconazole during the chronic stage of infection and then treated with cyclophosphamide (see the legend to Fig. 1). Images taken at 147 (upper) and 148 (lower) dpi. The schematic identifies the positions of organs. Gut Mes, gut mesentery tissue; OES, esophagus; SKM, skeletal muscle; STM, stomach; VIS FAT, visceral fat/adipose tissue. The location of the visceral fat tissue is highlighted by an arrow. Heat maps are on log_10_ scales and indicate the intensity of bioluminescence from low (blue) to high (red).

In 9 of the 16 posaconazole-treated mice that relapsed after cyclophosphamide treatment, we observed that adipose tissue was the major site of recrudescence (see, for example, Fig. 7A). This suggests that the ability of parasites to persist in this location following acute stage posaconazole treatment is a common phenomenon. In contrast (*P* < 0.05), when mice in the chronic stage of infection were treated and then immunosuppressed, only 1 of 9 animals displayed a significant parasite burden in this tissue (shown Fig. 1Eiv), with the gastrointestinal tract being the major site of parasite recrudescence (Fig. 7B).

## DISCUSSION

Progress in developing new drugs for chronic T. cruzi infections has been limited by difficulties in unambiguously demonstrating parasitological cure. An underlying cause, as inferred from murine infections, could be the discrete nature of infection foci during chronic Chagas disease and their highly dynamic spatiotemporal distribution (24). As a consequence, there is a risk of overestimating cure rates associated with unguided tissue sampling, even when using PCR-based technology. Highly sensitive bioluminescence imaging circumvents some of these issues by facilitating the evaluation of parasite burden throughout long-term infections, with minimal tissue sampling bias.

Several studies have reported on the efficacy of the CYP51 inhibitor posaconazole, and its ability to cure T. cruzi infections in murine models (17–20). Despite this, when the drug was advanced into clinical trials, it failed to provide significant benefit to chronically infected patients, in terms of parasitological cure (21). In line with this, data from our predictive model imply that posaconazole has limited potential against both stages of Chagas disease (Table 1). *In vivo* imaging revealed that although posaconazole is highly effective at reducing parasite burden, it does not readily cure acute or chronic T. cruzi infections. When mice in the acute stage were treated, the bioluminescence-inferred parasite burden was reduced by >3 orders of magnitude within 7 days; however, sterile cure was rarely achieved (Fig. 2 and 6). With chronic infections, posaconazole failed to cure any of the mice and the reduction in parasite load occurred more slowly (Fig. 1 and 2). This inability, in the vast majority of cases, to eradicate parasites has parallels with *in vitro* studies. These studies showed that although the 50% effective concentration of posaconazole against intracellular T. cruzi is in the nanomolar range, it often fails to eliminate parasite infection (22). One reason for the faster rate of parasite knockdown in the acute stage (Fig. 2) might be that parasites replicate more rapidly and are therefore more susceptible to drugs that perturb lipid metabolism. Alternatively, in the chronic stage, when parasites are restricted predominantly to gastrointestinal sites (24), they may be less accessible compared to the acute stage, when parasites can be targeted in all organs, although this explanation is less likely given the pharmacokinetic and distribution properties of posaconazole (30).

Typically, treatment with posaconazole reduced whole-body bioluminescence to background levels, with few infection foci detectable in the absence of immunosuppression. Given the sensitivity of the imaging procedure (24), this suggests that the remaining parasites survive in low numbers within small groups of infected cells. As a result, detection of residual parasites by PCR-based methods is problematic (Fig. 3). In the past, this may have led to an overestimation of the ability of posaconazole to cure chronic infections. Posaconazole treatment has been shown to reverse spleen enlargement, a characteristic of murine T. cruzi infections. In these experiments, curative outcome was inferred on the basis of several criteria (27). However, here we demonstrate that reversal of splenomegaly is not indicative of sterile cure (Fig. 5) but is linked merely with a reduction in parasite burden.

In more than 50% of cases (9/16), endpoint *ex vivo* analysis of acute-stage infections identified visceral fat as the tissue with the highest parasite burden following relapse (Fig. 7). There are several reasons why posaconazole could be less effective at eliminating parasites from this site. Parasite load may be higher in adipose tissues (24), differential drug accessibility may be an issue, or parasites could be less susceptible in a lipid/sterol rich environment. When mice treated during the chronic stage were examined after relapse, only one mouse of nine displayed a detectable level of bioluminescence in visceral fat (Fig. 7). At this stage of an infection, parasites are restricted mainly to the gastrointestinal tract, and only sporadically detected in the visceral fat, or other tissues, by bioluminescence. In chronic infections, therefore, this tissue is less likely to be relevant as a reservoir for parasite survival following drug treatment. Previous studies have identified parasites localized in adipose tissue in some chronic human infections (31, 32). In untreated mice, however, bioluminescence imaging did not identify the visceral fat as a primary site of recrudescence during a chronic infection (see Fig. 7).

In summary, we have shown that benznidazole is significantly more effective at curing both acute and chronic T. cruzi infections than posaconazole. The utility and flexibility of the *in vivo* imaging procedure we developed has potential for making a valuable contribution to the Chagas disease drug discovery pipeline. It can also, as shown here, add value to the screening process by providing new information on drug efficacy. Importantly, the availability of such a sensitive *in vivo* technique should provide greater assurance that drugs are not advanced prematurely into clinical trial.
